# High-density transposon libraries utilising outward-oriented promoters identify mechanisms of action and resistance to antimicrobials

**DOI:** 10.1093/femsle/fnaa185

**Published:** 2020-11-13

**Authors:** Chris Coward, Gopujara Dharmalingham, Omar Abdulle, Tim Avis, Stephan Beisken, Elena Breidenstein, Natasha Carli, Luis Figueiredo, David Jones, Nawaz Khan, Sara Malara, Joana Martins, Nabeetha Nagalingam, Keith Turner, John Wain, David Williams, David Powell, Clive Mason

**Affiliations:** Summit Therapeutics plc, The Merrifield Centre, 12 Rosemary Lane, Cambridge, CB1 3LQ, UK; Summit Therapeutics plc, The Merrifield Centre, 12 Rosemary Lane, Cambridge, CB1 3LQ, UK; Summit Therapeutics plc, The Merrifield Centre, 12 Rosemary Lane, Cambridge, CB1 3LQ, UK; Summit Therapeutics plc, The Merrifield Centre, 12 Rosemary Lane, Cambridge, CB1 3LQ, UK; Summit Therapeutics plc, The Merrifield Centre, 12 Rosemary Lane, Cambridge, CB1 3LQ, UK; Summit Therapeutics plc, The Merrifield Centre, 12 Rosemary Lane, Cambridge, CB1 3LQ, UK; Summit Therapeutics plc, The Merrifield Centre, 12 Rosemary Lane, Cambridge, CB1 3LQ, UK; Summit Therapeutics plc, The Merrifield Centre, 12 Rosemary Lane, Cambridge, CB1 3LQ, UK; Summit Therapeutics plc, The Merrifield Centre, 12 Rosemary Lane, Cambridge, CB1 3LQ, UK; Summit Therapeutics plc, The Merrifield Centre, 12 Rosemary Lane, Cambridge, CB1 3LQ, UK; Summit Therapeutics plc, The Merrifield Centre, 12 Rosemary Lane, Cambridge, CB1 3LQ, UK; Summit Therapeutics plc, The Merrifield Centre, 12 Rosemary Lane, Cambridge, CB1 3LQ, UK; Summit Therapeutics plc, The Merrifield Centre, 12 Rosemary Lane, Cambridge, CB1 3LQ, UK; Quadram Institute, Rosalind Franklin Road, Norwich Research Park, Norwich, NR4 7UQ, UK; Quadram Institute, Rosalind Franklin Road, Norwich Research Park, Norwich, NR4 7UQ, UK; Nanna Therapeutics, The Merrifield Centre, 12 Rosemary Lane, Cambridge, CB1 3LQ, UK; Summit Therapeutics plc, The Merrifield Centre, 12 Rosemary Lane, Cambridge, CB1 3LQ, UK; Summit Therapeutics plc, The Merrifield Centre, 12 Rosemary Lane, Cambridge, CB1 3LQ, UK

**Keywords:** bacteria, antibiotic, development, resistance, mechanism, mutagenesis

## Abstract

The use of bacterial transposon mutant libraries in phenotypic screens is a well-established technique for determining which genes are essential or advantageous for growth in conditions of interest. Standard, inactivating, transposon libraries cannot give direct information about genes whose over-expression gives a selective advantage. We report the development of a system wherein outward-oriented promoters are included in mini-transposons, generation of transposon mutant libraries in *Escherichia coli* and *Pseudomonas aeruginosa* and their use to probe genes important for growth under selection with the antimicrobial fosfomycin, and a recently-developed leucyl-tRNA synthase inhibitor. In addition to the identification of known mechanisms of action and resistance, we identify the carbon–phosphorous lyase complex as a potential resistance liability for fosfomycin in *E. coli* and *P. aeruginosa*. The use of this technology can facilitate the development of novel mechanism-of-action antimicrobials that are urgently required to combat the increasing threat worldwide from antimicrobial-resistant pathogenic bacteria.

## Teaser

The authors develop a mutagenesis procedure for use in the identification of antimicrobial mechanisms of action and resistance and identify a potential resistance mechanism for fosfomycin.

## INTRODUCTION

Antimicrobial resistance is an urgent and growing problem worldwide—with nearly 3M infections and ∼40 000 deaths annually in the US (CDC 2019). To combat this issue, there is an urgent need to develop novel antimicrobial agents with potent activity and low potential for resistance. Two main approaches are used to identify new agents: phenotypic screens, where antimicrobial compounds are identified by their ability to inhibit the growth of, or kill, bacteria; and target-based strategies where compounds are identified that can specifically inhibit essential bacterial proteins (Butler and Cooper 2012). To guide drug development, when phenotypic screens identify an active compound, it is important to identify its mechanism(s) of action, and any route(s) to resistance against the compound. Information on both these aspects can be gained by analysis of resistant mutant bacteria that arise either spontaneously or from transposon mutagenesis experiments.

**Figure 1.**
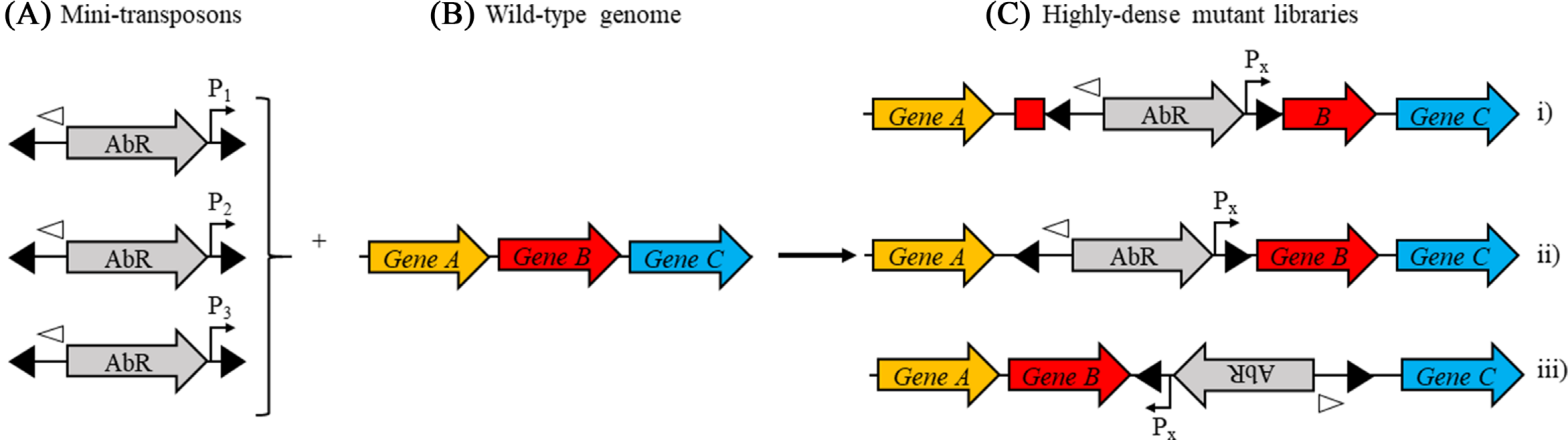
Overview of the technology. **(A)** Mini-transposons containing an antibiotic resistance cassette (AbR), IS*50* inverted repeats (filled triangles), different-strength outward-oriented promoters (P_1_, P_2_ and P_3_) and a binding site for sequencing the insert locations (open triangle) are introduced into; **(B)** The genome of the target organism, yielding; **(C)** Highly-dense mutant libraries wherein expression of random genes (in this case *geneB*) can be altered in one of three ways; (i) insertional inactivation; (ii) constitutive expression by insertion of the outward-oriented promoter (P_x_; expression level depends on the particular promoter inserted) upstream of the gene; or (iii) reduced expression by antisense inhibition when P_x_ is oriented towards the gene.

The use of mobile DNA elements—such as transposons (Tns)—for the isolation of bacterial mutants with specific phenotypes has a long history. Early experiments using signature-tagged mutagenesis (Hensel *et al*. 1995) relied on analysis of individually-isolated mutants able to survive a selective screen. The advent of microarrays (Chaudhuri *et al*. 2009) allowed the analysis of thousands of mutants, then next-generation sequencing technology enabled the measurement of fitness of mutants within pools of millions by methods such as TraDIS (Langridge *et al*. 2009) and TnSeq (van Opijnen, Bodi and Camilli 2009). These methods rely on insertion of Tns causing a phenotypic change by inactivation of genes. In nature however, Tn insertion can also activate gene expression—by transcription from a promoter within the Tn, or the formation of a mosaic promoter from Tn and chromosomally-located elements (Mahillon and Chandler 1998; Vandecraen *et al*. 2017). We have improved existing screening technologies by incorporating panels of outward-oriented promoters of differing strength into mini-transposons which are then used to generate high insertion-density libraries (∼1 M total mutants). The expression level of each gene is a product of the strength and location of the exogenous promoter. In principle, in these libraries, expression of every gene could be increased by different levels. In this way, phenotypic changes could be effected by: (i) disruption of gene function by insertion within the Open Reading Frame (ORF; Vandecraen *et al*. 2017); (ii) constitutive activation of genes by insertion 5′ to the ORF with the outward-oriented promoter facing the gene (Vandecraen *et al*. 2017) and (iii) down-regulation of genes by expression of antisense mRNA (Shearwin, Callen and Egan 2005; Saberi *et al*. 2016) following transposon insertion 3′ with the outward-oriented promoter directing transcription back towards the gene (Fig. 1). This approach has been used to investigate antimicrobial susceptibility in *Staphylococcus aureus* (Wang *et al*. 2011; Santiago *et al*. 2018), to identify genes involved in surviving exposure to triclosan in *Escherichia coli* (Yasir *et al*. 2019) and we have employed it during development of an anti-Enterobacteriaceae compound series.

In the context of antimicrobial research, these transposon libraries can be used in two main ways: determining candidate essential genes (those for which transposon insertion within the gene is not tolerated) to identify potential antimicrobial targets for target-based screens; and deciphering resistance mechanisms and mechanism of action for compounds identified in phenotypic screens. In the latter case, fitness differences between mutants can be quantified following exposure of the mutants to antimicrobial compounds. Increased fitness could be a consequence of: inactivation or down-regulation of a compound entry mechanism (e.g. a porin) or non-essential target; increased expression of a target protein; upregulation of mechanisms that hinder entry or promote efflux of the compound. Increased expression could be achieved either directly—by transcription from the outward-oriented promoters—or indirectly by modulation of regulatory systems (e.g. by repressor inactivation). Insertions could also reduce fitness in a compound-specific way by decreasing the expression of genes that confer a fitness advantage (e.g. an efflux mechanism) or increasing expression of those conferring a disadvantage (e.g. a porin required for compound entry). In addition to their utility in antimicrobial research, other applications of this technology are also possible—for example modifying gene expression for metabolic engineering or the induction of normally-cryptic loci for the production of secondary metabolites (Zarins-Tutt *et al*. 2016).

Here we report the validation of two outward-oriented promoter libraries constructed in *E. coli* BW25113 and *Pseudomonas aeruginosa* NCTC#11451, and demonstrate proof-of-concept data for target and resistance mechanism identification with a clinically-used antibiotic (fosfomycin). We also apply the technology to a recently-developed bacterial leucyl-tRNA synthetase inhibitor (GSK2251052), development of which was suspended in a phase 2 clinical trial due to high levels of spontaneous resistance.

## RESULTS AND DISCUSSION

### Generation of high-density transposon libraries with outward-oriented promoters

We designed and constructed a panel of mini-Tn*5* transposons containing an antibiotic resistance cassette for selection of mutants and different promoters oriented towards one terminal inverted repeat to drive transcription out of the mini-transposon (Fig. 1). Transposon mutant libraries were generated in *E. coli* BW25113 and *P. aeruginosa* NCTC#11451. Determination of the sites of transposon insertion in these libraries showed that they were of high-density, containing 100 000s of insertion sites per promoter (Table 1). Mean insertion site frequency per base pair for standard transposon libraries is determined by dividing the genome size by the number of insertion sites. For our transposons, the direction of insertion is also important as transcription from the outward-oriented promoter proceeds in only one direction, so insertion frequency was calculated by dividing twice the genome size by the number of insertion sites (frequency=[genome size x 2]/[no. insertion sites]).

**Table 1.** Mini-transposon libraries constructed in this study for *E. coli* (Ec) and *P. aeruginosa* (Pa).

				For each promoter	
Strain	Resistance cassette	Selection^1^	Outward-oriented promoters^2^	No. insertion sites	Insertion site frequency (b.p. per insertion)
Ec	*aph(3)-I* ^4^	Km	PrplJ; PrrnB; Ptac	∼6.0 × 10^5^	∼15
Pa	*aac(3)-I* ^5^	Gm	PrplJ; PrrnB; Ptac; PsPrrnB	∼5.0 × 10^5^	∼26

1 Antibiotic used when creating the mutant libraries, Km: kanamycin; Gm: gentamcin.

2 Promoter sequences are in the Supplementary Material.

3 Calculated using genome size 4.6 Mb for Ec, 6.5 for Pa.

4 Synthesised.

5 From pFastBact1.

### Proof-of-concept for antimicrobial target and resistance mechanism identification using fosfomycin in *E. coli* and *P. aeruginosa* and GSK2251052 in *E. coli*

Fosfomycin is a broad-spectrum antibiotic in use since the 1970s primarily for treatment of urinary-tract infections (UTI). There has been renewed interest recently in its use for other indications due to its activity against multidrug-resistant pathogens but whether clinical resistance will compromise its use is unclear (Silver 2017). Fosfomycin exerts its bactericidal activity by inhibiting cell wall biogenesis via inhibition of UDP-*N*-acetylglucosamine-enolpyruvyltransferase (MurA) which catalyses the first committed step of peptidoglycan synthesis (Silver 2017). *In vitro* resistance is primarily driven by mutations in the transport systems required for uptake of fosfomycin (GlpT: responsible for α-glycerophosphate uptake; and UhpT which transports phosphates and is induced by glucose-6-phosphate [G6P]) (Silver 2017). Mutations in, or overexpression of, *murA* have been shown to result in resistance (Wu and Venkateswaran 1974; Takahata *et al*. 2010; Couce *et al*. 2012). In *E. coli*, *glpT* and *uhpT* are positively regulated by cAMP and fosfomycin sensitivity can decrease in strains with mutations in *cyaA* (adenylate cyclase, which catalyses the formation of cAMP) and *ptsI* (part of the PTS system required for expression of *cyaA*; Silver 2017). Resistance to fosfomycin can also be acquired by expression of fosfomycin-modifying enzymes (Silver 2017). These well-defined resistance mechanisms, including both gene disruption and overexpression, made fosfomycin an ideal compound to validate our system—indeed we successfully identified all of these mechanisms, as described below (this technique can of course not determine resistance mechanisms acquired by lateral transfer of genetic material).

We tested the utility of our technology to identify mechanisms of resistance to, and action of, two antimicrobials. First, we exposed *E. coli* mutant libraries to sub- and supra-MIC concentrations (0.25 ×, 0.5 ×, 0.75 ×, 1 ×, 2 × and 4 × MIC) of fosfomycin with G6P (to induce expression of UhpT thus increasing sensitivity to the compound), and mapped the sites of transposon insertion in surviving bacteria. There was a concentration-dependent decrease in the number of mapped insertion sites (Table S1, Supporting Information), clearly representing bacteria that were capable of growth under selection. We classified genes as Disrupted, Activated or with Complex patterns of insertions under selection (see the Methods for classification criteria and Supplementary Material S1–S3 for genes affected).

**Figure 2.**
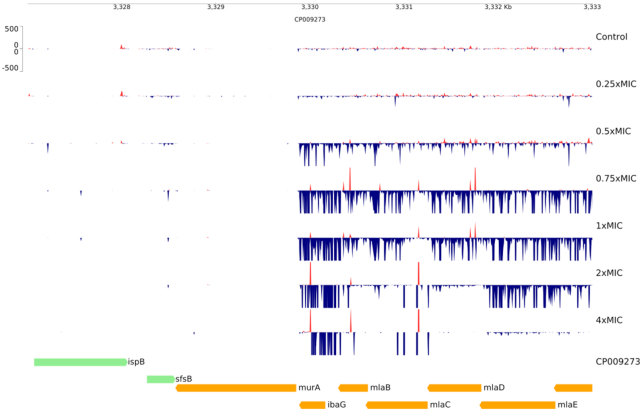
Selection for activating insertions upstream of *murA* during exposure of *E. coli* BW25113 libraries to fosfomycin in the presence of G6P (25 µg/mL). Fosfomycin concentration is shown as multipliers of the MIC (3.125 µM). Insertion locations are shown as peaks coloured to indicate the orientation of the outward-facing promoter with respect to the genome sequence; red: 5′ to 3′ on the top strand; blue: 5′ to 3′ on the bottom strand. Genes are displayed below the insertion plots and coloured by their direction of transcription with respect to the genome sequence; green: 5′ to 3′ on the top strand; yellow: 5′ to 3′ on the bottom strand.

Where increased expression of an antibiotic target can reduce sensitivity, which is known to be the case for fosfomycin (Couce *et al*. 2012), we would expect to see activating signals upstream of the gene encoding the target. We observed this for *murA* at all concentrations—with up to a 17 log_2_-fold increase above control (Fig. 2 and Supplementary Material S1). Notably, at lower fosfomycin concentrations, activating insertions were found throughout the upstream *mlaFEDCB* operon whereas at higher concentrations only insertions within ∼600 b.p. upstream of *murA* were observed. In total, two potentially-compatible hypotheses could explain this observation: (i) insertion of any of the outward-oriented promoters further from *murA* could not increase expression enough to give a selective advantage at these compound levels and (ii) disruption of the *mlaFEDDCB* operon—encoding a transport system required for maintenance of outer membrane lipid asymmetry (Malinverni and Silhavy 2009)—could confer a selective disadvantage in the presence of higher levels of fosfomycin. Disruption of these genes does not result in a general growth defect but is disadvantageous when the outer membrane lipopolysaccharide is destabilised (Malinverni and Silhavy 2009).

Disruption of expression of the UhpT transporter protein is a known resistance mechanism. UhpT is expressed from the *uhpABCT* operon, where UhpA is the transcriptional activator, phosphorylated by the membrane-associated signal transduction complex UhpBC. We observed disruption signals in this operon which would be predicted to prevent UhpT expression. For *uhpA, C* and *T*, disruption (up to 12-, 11- and 12 log_2_-fold over control, respectively) was unambiguous, with insertions on both strands throughout the ORFs (see Figure S1, Supporting Information and Supplementary Material S1). For *uhpB*, insertions (up to 9 log_2_ over control) were observed predominately in the 5′ half of the gene, oriented such that the outward-transcribing promoter would yield transcription towards *uhpA*. This would be predicted to decrease expression of the UhpA activator via antisense RNA inhibition.

We also saw disrupting insertions within genes required for cAMP regulation (*cra, crr, crp*and*ptsHI*) and formation (*cya*), which is as expected since *uhpT* expression is positively regulated by cAMP and disruption of cAMP expression is known to result in resistance to fosfomycin (Silver 2017). The protein domain organisation of *cya* was clearly reflected in the pattern of insertions (Figure S2, Supporting Information and Supplementary Material S1): at the highest concentration, selection was for insertions in the 5′ half of the gene—which contains the catalytic domain (Crasnier, Dumay and Danchin 1994). Disruption of this domain would prevent formation of cAMP, reducing *glpT* and *uhpT* expression thus giving a selective advantage. As fosfomycin concentration decreased, insertions in the 3′ half were observed, primarily with the outward-transcribing promoter facing the 5′ end of the gene. The C-terminal domain exerts negative regulation on the catalytic domain and disruption of this regulatory domain increases adenylate cyclase activity (Crasnier, Dumay and Danchin 1994), resulting in a selective disadvantage. However, expression from the outward-transcribing transposon promoter may decrease catalytic domain expression via antisense inhibition. At even lower fosfomycin concentrations (1 × MIC and below), bidirectional insertions were seen both at the extreme 3′ end of the gene and immediately upstream (in the promoter region). The insertions upstream of the gene likely dysregulate *cya* expression, giving a selective advantage. It is unclear why disruption of the extreme 3′ end of the gene would be selected at these concentrations.

**Figure 3.**
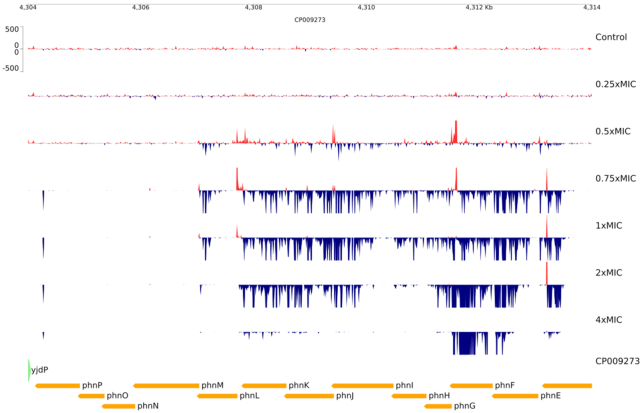
Selection for mini-transposon insertions in the *phn* operon during exposure of *E. coli* BW25113 libraries to fosfomycin in the presence of G6P (25 µmg/mL). Fosfomycin concentration is shown as multipliers of the MIC (3.125 µM). Insertion locations are shown as peaks coloured to indicate the orientation of the outward-facing promoter with respect to the genome sequence; red: 5′ to 3′ on the top strand; blue: 5′ to 3′ on the bottom strand. Genes are displayed below the insertion plots and coloured by their direction of transcription with respect to the genome sequence; green: 5′ to 3′ on the top strand; yellow: 5′ to 3′ on the bottom strand.

As well as known resistance mechanisms we could identify other loci whose mutation resulted in a selective advantage for *E. coli*. There was a complex pattern of insertions in the *phn* operon (*phnC-phnP*; Fig. 3 and Supplementary Material S1) encoding the enzymes of the CP-lyase pathway which cleaves carbon-phosphorous bonds of a variety of substrates and is upregulated under conditions of phosphate starvation (Kamat and Raushel 2013; Stosiek, Talma and Klimek-Ochab 2019). With fosfomycin at the highest tested concentrations, strong unidirectional insertion signals were observed in *phnF*—the transcriptional repressor of the *phn* operon—with the mini-transposon outward-transcribing promoter oriented towards *phnG*. Such insertions are predicted to increase expression of the operon thus resulting in increased CP-lyase activity. While a direct role for this operon in degradation of fosfomycin has not been reported, bacteria with broad-spectrum CP-lyase activity can use fosfomycin as a sole carbon and phosphorous source (Wacket *et al*. 1987; Quinn 1989). At lower concentrations, unidirectional inserts were observed throughout the operon 5′ to *phnM*. Insertions 5′ to *phnF* would result in constitutive expression of the operon, despite potentially increasing expression of the repressor, as activity of the mini-transposon outward-transcribing promoter would be unaffected by PhnF-mediated repression. Insertions within *phnGHIJK* are predicted to abolish the ability of the bacteria to cleave the C–P bond but they must still confer a selective advantage for growth, presumably by increasing expression of *phnMNOP* (within which insertions were not selected for), although the mechanism for this advantage is unclear. The *phn* operon is part of the *pho* regulon (Stosiek, Talma and Klimek-Ochab 2019), expression of which can be rendered constitutive by mutation in the *pst* operon (Wanner 1993) within which we observed selection for inactivating insertions (Supplementary Material S1). These results are consistent with those published in a recent study using transposon-mediated insertion of a regulated promoter (Turner *et al*. 2020).

Increased expression of the *phn* operon is clearly tolerated *in vitro* as seen by the large number of inserts found in its repressor even in the control samples. If the same is true *in vivo* during infection there is a clear risk that repressor mutations could result in increased resistance to fosfomycin, although such mutations have not as yet been observed under fosfomycin selection *in vitro* or *in vivo*.

**Figure 4.**
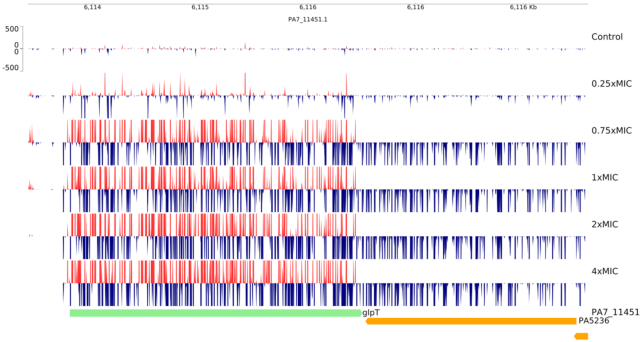
Selection for mini-transposon insertions at *glpT* after exposure of *P. aeruginosa* NCTC#11451 libraries to fosfomycin. Shows both disruption of *glpT* and antisense inhibition of *glpT* from insertions in *PA5236* with the mini-transposon outward-oriented promoters facing the 3′ end of *glpT*. Fosfomycin concentration is shown as multipliers of the MIC (12.5 µM). Insertion locations are shown as peaks coloured to indicate the orientation of the outward-facing promoter with respect to the genome sequence; red: 5′ to 3′ on the top strand; blue: 5′ to 3′ on the bottom strand. Genes are displayed below the insertion plots and coloured by their direction of transcription with respect to the genome sequence; green: 5′ to 3′ on the top strand; yellow: 5′ to 3′ on the bottom strand.

We also tested libraries made in *P. aeruginosa* NCTC#11451 (Table S2, Supporting Information and Supplementary Material S2 for genes affected). This organism does not possess a G6P-inducible permease—fosfomycin gains entry into the cell via the GlpT permease (Castañeda-Garcia *et al*. 2009). As expected, disrupting signals were observed in *glpT* (up to 15 log_2_-fold over control). Strikingly there were also strong unidirectional insertions in *PA5236*, predicted to encode CDP-6-deoxy-delta-3,4-glucoseen reductase, which is convergently transcribed to *glpT* (Fig. 4). These insertions likely give a selective advantage due to antisense inhibition of *glpT* expression. Unlike in *E. coli*, selection for activation of *murA* was not observed. Possibly overexpression of *murA* does not confer a selective advantage in the presence of fosfomycin in *P. aeruginosa*. Alternatively, increased *murA* expression may be somewhat deleterious in this organism—reducing any selective advantage that *murA* over-expressors may have in the presence of fosfomycin, especially as compared to the *glpT*-disrupted mutants. We also observed selection for insertions in the *phn* operon with a pattern distinct from that in *E. coli* (Figure S3, Supporting Information**)**. In *P. aeruginosa* selection for bidirectional insertions was observed throughout *phnCDEFGHIJKL* with the major signals in *phnCDEF* showing a bias for orientation with the outward-transcribing promoter driving expression from *phnG* onwards. The genes *phnCDE* encode phosphonate transporters (Kamat and Raushel 2013) but this uptake system is not functional in *E. coli* K strains (Makino *et al*. 1991)—including *E. coli* BW25113 used in this study. The strong selection for disrupting insertions in the *P. aeruginosa phnCDEF* genes suggests fosfomycin may also gain entry to the cell by this transport system. Insertions in *phnMNP* (*phnO* is absent in *P. aeruginosa*), as in *E. coli*, were not selected for under fosfomycin selection.

Other genes with strong selection included activating insertions for *glpD* (glycerol-3-phosphate dehydrogenase)—which could suggest an interaction between *glpD* and *glpT* expression—and for insertions in the *PA4917-PA4916* (*nadD2-ntrR*) operon. The significance of this operon in fosfomycin sensitivity is not clear; although *nadD2-ntrR* have recently been shown to affect type III secretion via cAMP-signalling in *P. aeruginosa* (Jin *et al*. 2019) we are not aware of any evidence that such signalling affects *glpT* expression in this organism.

**Figure 5.**
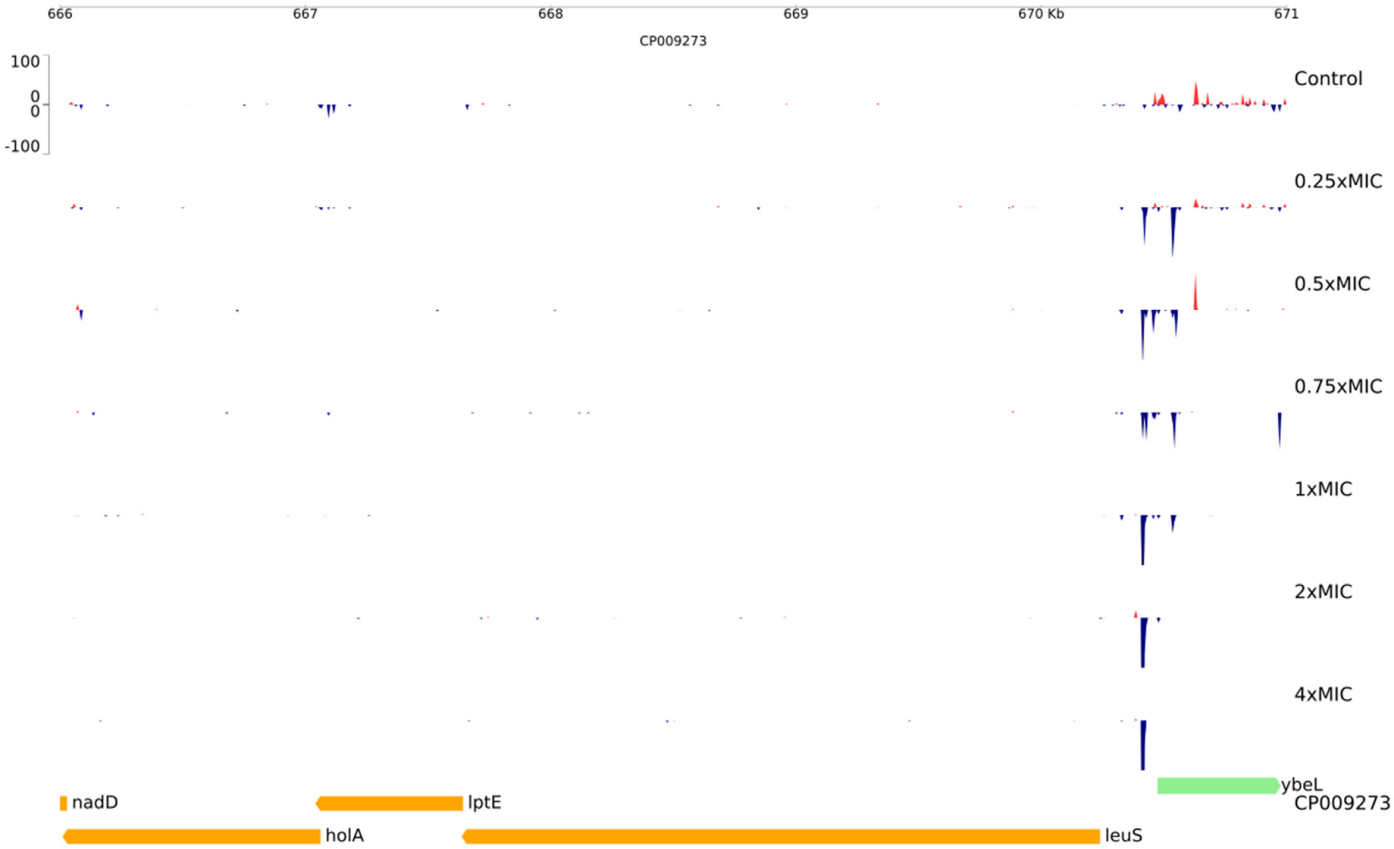
Selection for insertions 5′ to *leuS* after exposure of *E. coli* BW25113 libraries to GSK2251052. Shows activation of *leuS* due to insertions oriented such that the mini-transposon outward-facing promoter can drive expression of *leuS*. GSK2251052 concentration is shown as multipliers of the MIC (12.5 µM). Insertion locations are shown as peaks coloured to indicate the orientation of the outward-facing promoter with respect to the genome sequence; red: 5′ to 3′ on the top strand; blue: 5′ to 3′ on the bottom strand. Genes are displayed below the insertion plots and coloured by their direction of transcription with respect to the genome sequence; green: 5′ to 3′ on the top strand; yellow: 5′ to 3′ on the bottom strand.

To further validate the methodology and assess its potential utility in antimicrobial discovery and development we profiled the leucyl-tRNA synthetase inhibitor GSK2251052, development of which was suspended during a phase 2 clinical trial for complicated UTI due to development of spontaneous high-level resistance through mutation of the LeuS target (O'Dwyer *et al*. 2015). We exposed our *E. coli* library to GSK2251052 and observed extremely strong selection (up to 20 log_2_-fold over control) for activating insertions upstream of the target-encoding *leuS* (Fig. 5 and Supplementary Material S3 for genes affected). Extremely strong selection at a single location can be viewed as a disadvantage for a compound as it suggests a potential target-based resistance liability that needs to be understood or mitigated to permit further development.

These observations confirm that by assessing the pattern of transposon insertions, our outward-transcribing transposon promoter system extends the usefulness of transposon mutant libraries in the characterisation of antibacterial compounds. In addition to the identification of candidate essential genes—useful for target-based approaches—we can: identify putative antimicrobial targets where overexpression leads to increased fitness upon compound exposure (exemplified by *murA* and *leuS*); identify known mechanisms of reduced susceptibility, including those that may contribute to clinical resistance; and identify potential new resistance liabilities. This method can be applied to pathogenic bacteria, both Gram-positive and Gram-negative, that are responsible for many of the resistance challenges that we face across our healthcare systems. By using this methodology at all stages of the drug discovery process we are accelerating candidate identification and lead optimisation by improving the understanding of compound mechanisms of action and resistance, as currently exemplified by an anti-Enterobacteriaceae series under active development.

## METHODS

### Bacterial strains and growth conditions

*E. coli* BW25113 and *P. aeruginosa* NCTC#11451 were routinely cultured at 37°C on L-agar plates or in L-broth with shaking.

### Preparation of mini-transposons

All mini-transposons consisted of IS*50* terminal inverted repeats (TIRs) flanking an antibiotic resistance cassette for selection placed 5′ to an outward-oriented promoter, and a binding site for a next-generation sequencing-compatible primer (schematic in Fig. 1). Resistance cassettes and promoters varied between strains (Table 1). Plasmids containing the mini-transposons were prepared as follows: for *E. coli*, pBluescript II (SK+) plasmids containing the mini-transposon with outward-oriented P*rplJ*, P*rrnB* or P*tac* were made by synthesis (MWG Biotech); for *P. aeruginosa*, derivatives containing an alternative selection cassette and outward-oriented promoters were constructed by standard cloning methods. Promoter sequences are in Table S3 (Supporting Information).

Mini-transposons for mutagenesis were generated from these plasmids by PCR using Q5® DNA polymerase (New England Biolabs, Hitchin, UK) with primers Tn5–03 (5′-ctgtctcttatacacatctccct) and Tn5–04 (5′-ctgtctcttatacacatctcttc)—which bind in the TIRs—and treated with polynucleotide kinase prior to purification by phenol:chloroform extraction, isopropanol precipitation with resuspension in 10 mM Tris-Cl (pH 8.5) and quantification by QuBit (ThermoFisher, Gloucester, UK). Template plasmid carry-over was prevented by restriction digestion of either the template plasmid with *Eci*I prior to PCR, or the PCR product with *Dpn*I prior to purification.

### Transposon mutagenesis

Transposon mutagenesis was by *in vivo* transposition following electroporation with mini-transposon:transposase complexes prepared *in vitro* (Goryshin *et al*. 2000), detailed methods are found in Supplementary Material Methods. Entire transformations were then plated onto L-agar with selection in 245 mm square assay plates and incubated overnight at 37°C. Bacteria were harvested from the plates into L-broth, glycerol added to 15% (w/v) and stored at −80°C. Typically at least 300 000 colonies were harvested per transposon with transformations repeated to achieve these numbers. To test whether this method of library generation yielded mutants with a single transposon insertion, whole-genome sequencing (WGS) of 20 individual *E. coli* mutants was performed—100% had a single transposon insertion. The number of individual mutants and insertion site frequency (Table 1) in the mutant libraries was estimated by determining the number of unique transposon sites in the pools by next generation sequencing (see below). Final combined library pools were made for each strain by mixing individual transposon pools normalised by A600nm measurement to contain approximately equal densities of each mutant.

### Selection of transposon mutants conferring growth advantage in the presence of antimicrobial compound

Libraries were screened in iso-sensitest broth (Oxoid) culture in duplicate 5 mL aliquots in 24-well plates. Compound concentrations varied between experiments but were typically chosen initially to cover 0.25 ×, 0.5 ×, 0.75 ×, 1 ×, 2 × and 4 × the compound MIC. Combined library pool sufficient for approximately 150–250 copies of each mutant/well was inoculated into 20 mL room-temperature compound-free media, incubated statically at 37°C for 20 min and then added to sufficient pre-warmed media to prepare the inoculum for all wells (final initial cell density was typically ∼10^8^ CFU/mL). A total of 5 mL aliquots were then dispensed into deep 24-well plates containing compound, plates covered with a breathable membrane and incubated with shaking (250 rpm) at 37°C for 16 h. Cultures were passaged once overnight by diluting 1:100 into pre-warmed media containing the same concentrations of compound as the initial growth. Bacteria were harvested by centrifugation and stored at −80°C prior to DNA extraction and sequencing.

### Next generation sequencing for identification of mini-transposon insertion sites

Insertion sites were determined by next-generation sequencing on the IonTorrent (ThermoFisher) or Illumina (Cambridge, UK) platforms for the GSK2251052 and fosfomycin samples respectively. Detailed methods are found in the Supplementary Material Methods. In brief: cell pellets were lysed, DNA was extracted and fragmented. Fragmented DNA was then purified using KAPA Pure Beads (Roche, Welwyn Garden City, UK) prior to addition of platform-specific adaptors by PCR. Reaction products were purified with KAPA Pure Beads prior to addition of sample-specific barcodes by PCR. For IonTorrent, the reaction products were size-selected (see later); for Illumina, products were purified with KAPA Pure Beads prior to addition of indexing primers by PCR and size-selection. Size selection was by KAPA Pure Beads, selecting for 150 b.p. (IonTorrent) or 250–350 b.p. (Illumina).

Following size-selection, DNA was quantified by qPCR and sequenced on an Ion Torrent Proton or Illumina NextSeq 500 as appropriate.

### Mapping of mini-transposon insertion sites and data processing

Quality of the raw sequencing reads were assessed using FastQC (Andrews 2020). Using a PERL script, sequencing reads were trimmed to remove sequencing adapter sequences and the mini-transposon end (5′-AGATGTGTATAAGAGACAG-3′), leaving only genomic sequences corresponding to the insertion site in the genome. Trimmed reads less than 20 b.p. were discarded.

The trimmed reads were aligned to the reference genome (Ec: BW25113-Accession CP009273; Pa: NCTC#11451, sequenced in-house) using bwa (Li and Durbin 2009). SAM files were processed using a PERL script to: (1) Identify transposon insertion sites in the genome; (2) summarise the number of reads on the forward and reverse strands for each insertion site; and (3) to export a strand-specific plot file to visualise the genome wide insertion sites coverage using Artemis (Carver *et al*. 2012).

To identify genes important for growth under the selection pressure, the total number of sequencing reads that mapped to a gene were summarised over the ORF and conditionally-essential genes were identified by comparing read counts of compound-treated samples to control using DESeq2 (Love, Huber and Anders 2014). Genes with Benjamini–Hochberg adjusted *P*-value < 0.05 and log_2_FC > 2 were considered to be disrupted. We then used stringent criteria to select high-confidence Disrupted genes with at least three insertion sites with at least 30 reads per insertion sites for downstream analysis. Separately, reads were summarised on each strand for Activation inserts within 250 b.p. 5′ to the start of the ORF using the same criteria.

### Classification of transposon insertion effects on genes

Following determination of transposon insertion site and orientation, we initially identified genes for which insertions within the ORF gave a selective advantage (at least three insertion sites with 30 reads each, with a >2 log_2_ increase in insertions within the ORF in both replicates at any concentration). The pattern of insertions was then visualised in Artemis (Carver *et al*. 1985) and the genes classified as UnambiguouslyDisrupted if insertions in both orientations in both control and treated samples were present. The remaining genes were classified as ComplexDisrupted if there was a clear pattern of insertions within the gene but not in both orientations in both control and treatment, examples of this type include: a bias in insertion orientation in both sample and control (selective advantage could be due to gene disruption and/or promotion of a downstream gene); insertions in only part of the open reading frame (potentially evidence for protein domain organisation)—see Figure S4 (Supporting Information) for examples. There was a final set of genes classified as DisruptedUnassigned where a very small number of insertion sites were identified at high intensity in the ORF, this was particularly common for GSK2251052, presumably reflecting spontaneously-resistant bacteria, which arise at high frequency for this compound (O'Dwyer *et al*. 2015).

Next, for genes that had not been identified as Disrupted (either Unambiguously or Complex), we identified those with an increase in insertions within 250 b.p. 5′ to the gene with the outward-transcribing promoter driving expression of the gene. These were classified as Unambiguously Activated (insertions in both orientations in control but biased towards driving expression of the gene in the sample). The remaining genes were designated as ComplexAcitvated—examples include where: insertions were unidirectional in the direction of gene transcription in both sample and control; where insertions at increased intensity were bidirectional upstream; there was a single high-intensity peak upstream of the gene—see Figure S5 (Supporting Information) for examples. Again, in the case of GSK2251052 this last category contained many examples where there were single peaks, likely due to spontaneous resistance.

## Supplementary Material

fnaa185_Supplemental_Files
