# Supplementary material for: High-density transposon libraries utilising outward-oriented promoters identify mechanisms of action and resistance to antimicrobials
Source: FEMS Microbiol Lett. 2020 Nov 13;367(22):fnaa185. doi: 10.1093/femsle/fnaa185 (PMC7735965; doi:10.1093/femsle/fnaa185)
Supplement: fnaa185_Supplemental_Files [file fnaa185_supplemental_files.zip › SupplementaryMaterialFigures.pptx]

## Slide 1
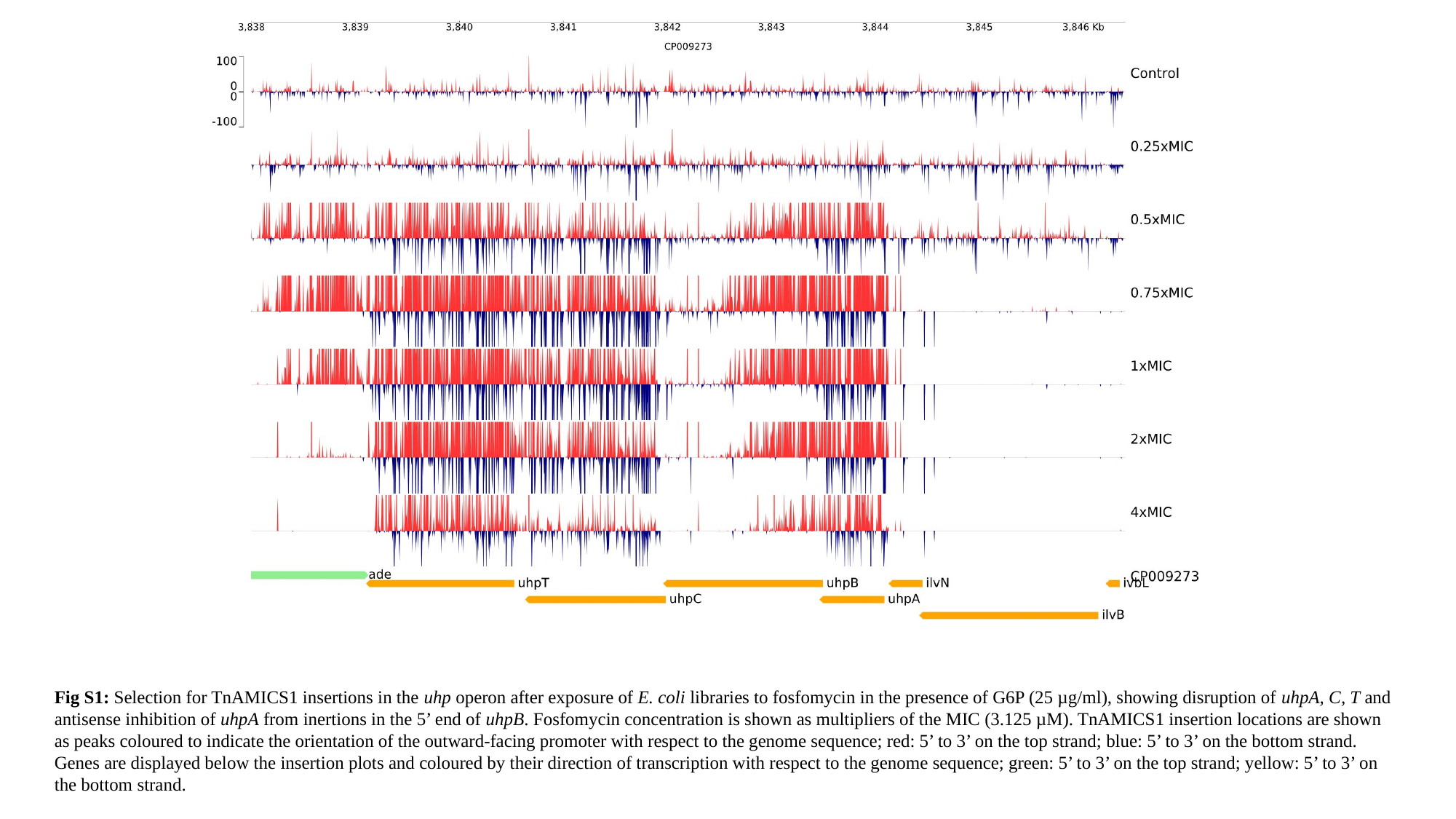

Fig S1: Selection for TnAMICS1 insertions in the uhp operon after exposure of E. coli libraries to fosfomycin in the presence of G6P (25 µg/ml), showing disruption of uhpA, C, T and antisense inhibition of uhpA from inertions in the 5’ end of uhpB. Fosfomycin concentration is shown as multipliers of the MIC (3.125 µM). TnAMICS1 insertion locations are shown as peaks coloured to indicate the orientation of the outward-facing promoter with respect to the genome sequence; red: 5’ to 3’ on the top strand; blue: 5’ to 3’ on the bottom strand. Genes are displayed below the insertion plots and coloured by their direction of transcription with respect to the genome sequence; green: 5’ to 3’ on the top strand; yellow: 5’ to 3’ on the bottom strand.

## Slide 2
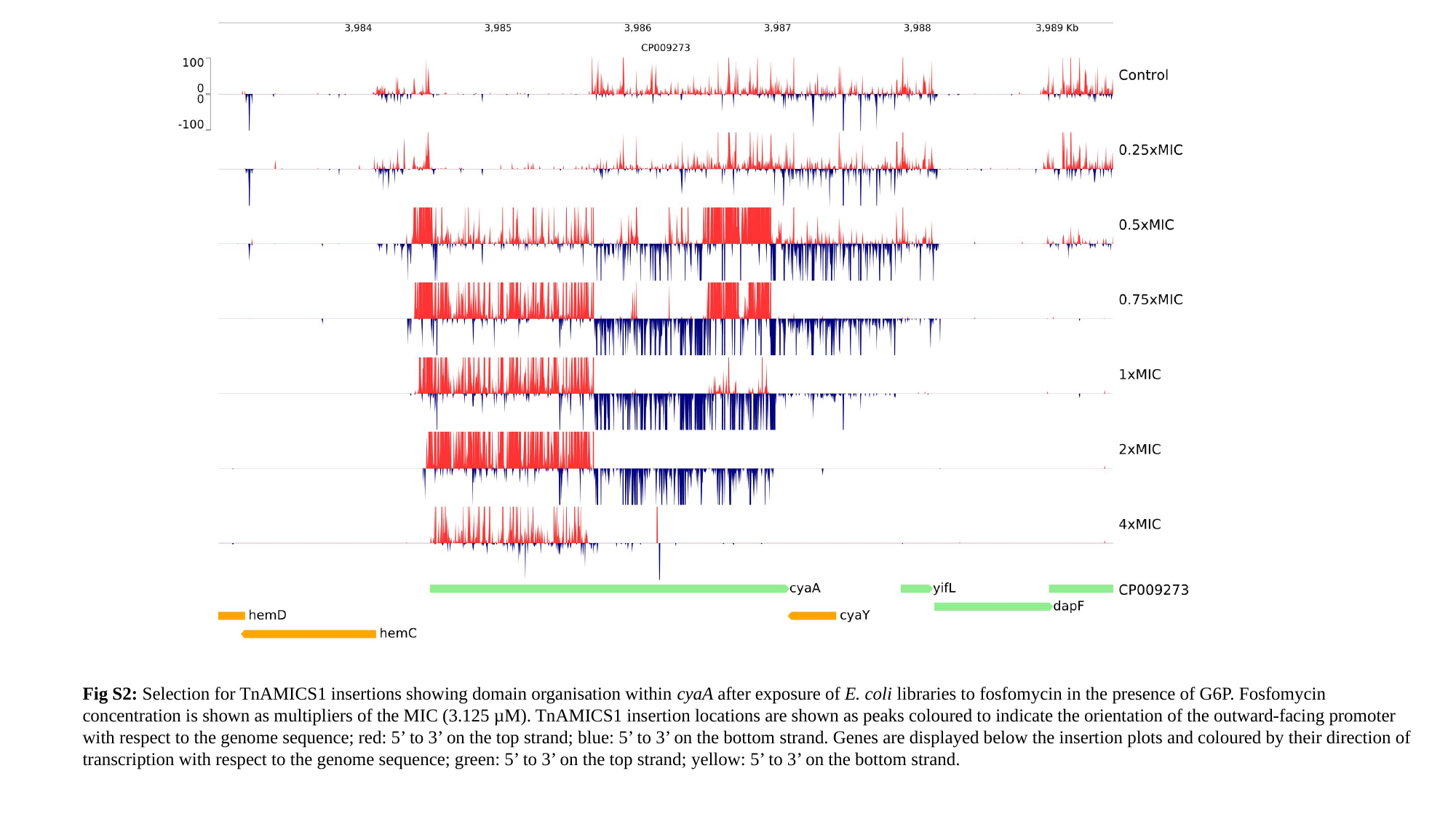

Fig S2: Selection for TnAMICS1 insertions showing domain organisation within cyaA after exposure of E. coli libraries to fosfomycin in the presence of G6P. Fosfomycin concentration is shown as multipliers of the MIC (3.125 µM). TnAMICS1 insertion locations are shown as peaks coloured to indicate the orientation of the outward-facing promoter with respect to the genome sequence; red: 5’ to 3’ on the top strand; blue: 5’ to 3’ on the bottom strand. Genes are displayed below the insertion plots and coloured by their direction of transcription with respect to the genome sequence; green: 5’ to 3’ on the top strand; yellow: 5’ to 3’ on the bottom strand.

## Slide 3
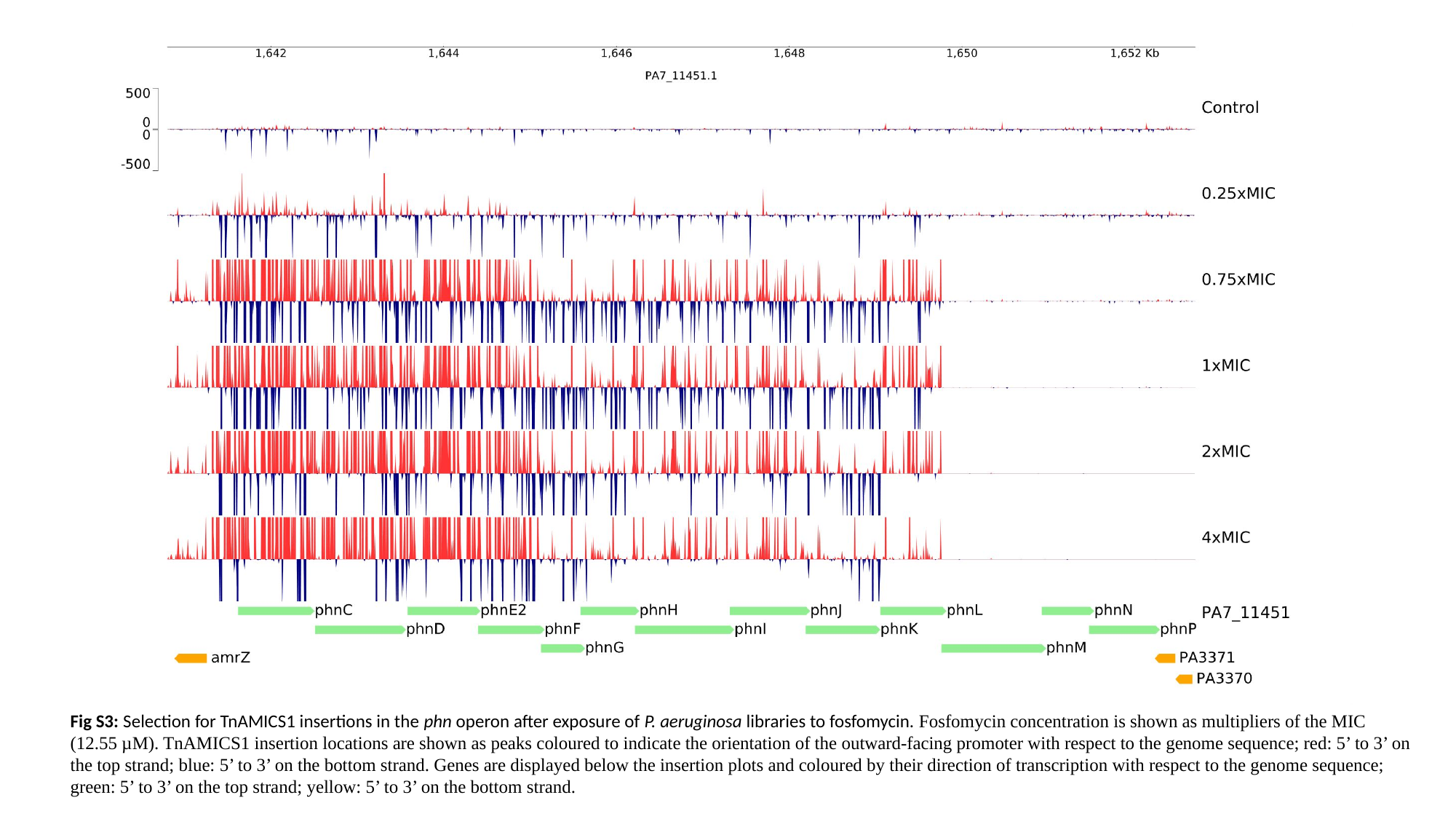

Fig S3: Selection for TnAMICS1 insertions in the phn operon after exposure of P. aeruginosa libraries to fosfomycin. Fosfomycin concentration is shown as multipliers of the MIC (12.55 µM). TnAMICS1 insertion locations are shown as peaks coloured to indicate the orientation of the outward-facing promoter with respect to the genome sequence; red: 5’ to 3’ on the top strand; blue: 5’ to 3’ on the bottom strand. Genes are displayed below the insertion plots and coloured by their direction of transcription with respect to the genome sequence; green: 5’ to 3’ on the top strand; yellow: 5’ to 3’ on the bottom strand.

## Slide 4
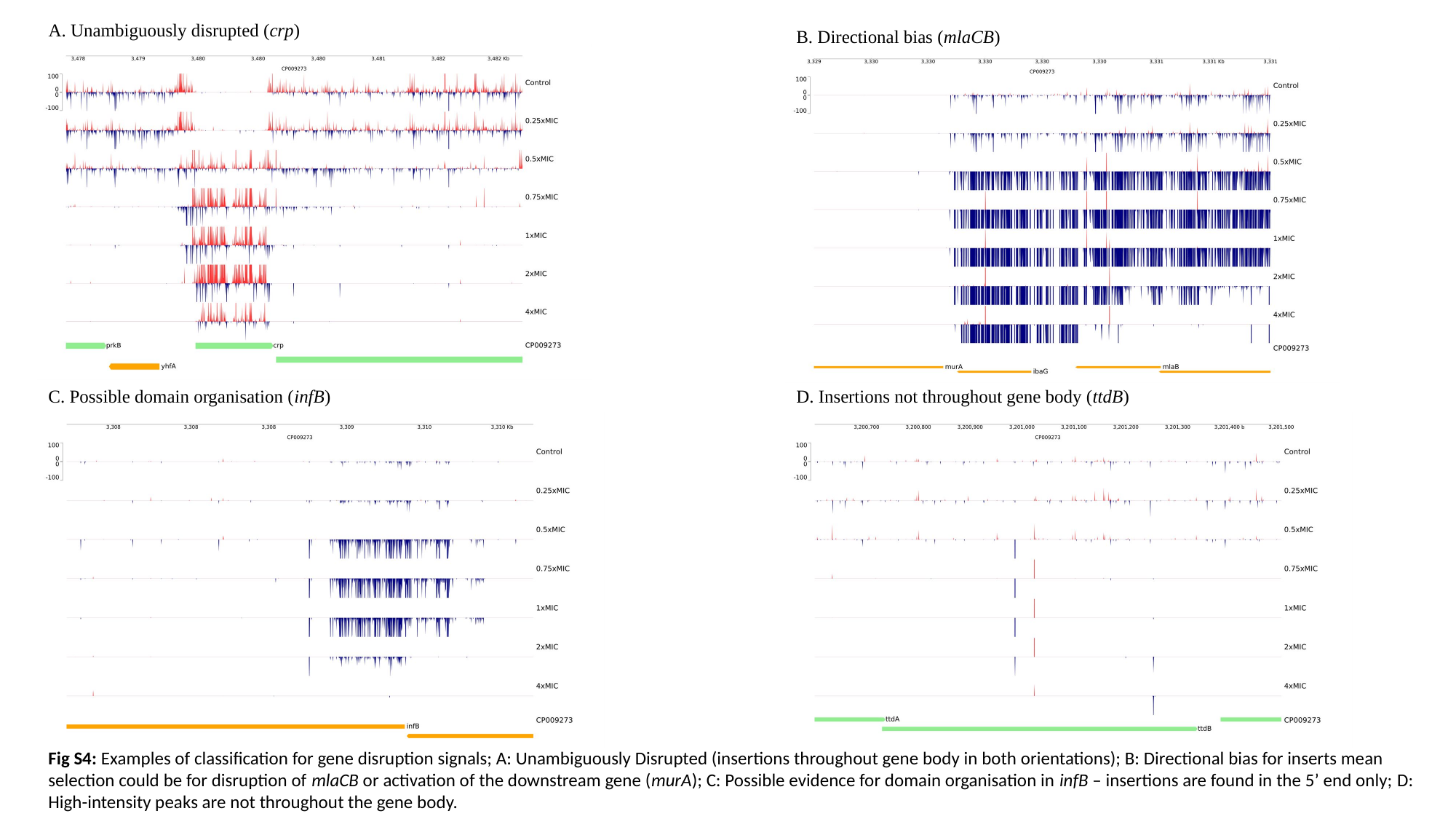

A. Unambiguously disrupted (crp)
B. Directional bias (mlaCB)
C. Possible domain organisation (infB)
D. Insertions not throughout gene body (ttdB)
Fig S4: Examples of classification for gene disruption signals; A: Unambiguously Disrupted (insertions throughout gene body in both orientations); B: Directional bias for inserts mean selection could be for disruption of mlaCB or activation of the downstream gene (murA); C: Possible evidence for domain organisation in infB – insertions are found in the 5’ end only; D: High-intensity peaks are not throughout the gene body.

## Slide 5
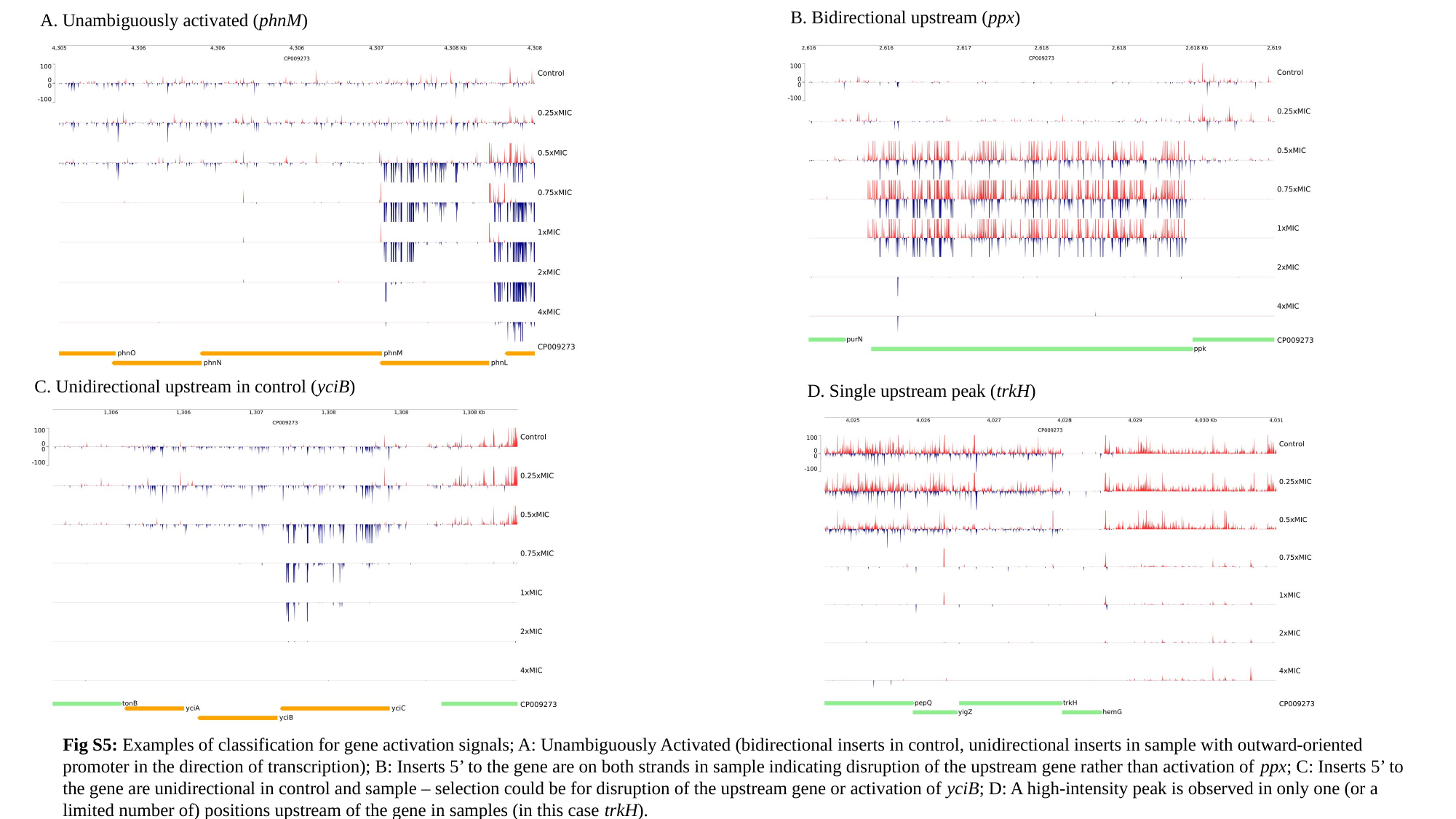

B. Bidirectional upstream (ppx)
A. Unambiguously activated (phnM)
C. Unidirectional upstream in control (yciB)
D. Single upstream peak (trkH)
Fig S5: Examples of classification for gene activation signals; A: Unambiguously Activated (bidirectional inserts in control, unidirectional inserts in sample with outward-oriented promoter in the direction of transcription); B: Inserts 5’ to the gene are on both strands in sample indicating disruption of the upstream gene rather than activation of ppx; C: Inserts 5’ to the gene are unidirectional in control and sample – selection could be for disruption of the upstream gene or activation of yciB; D: A high-intensity peak is observed in only one (or a limited number of) positions upstream of the gene in samples (in this case trkH).
