# Supplementary material for: High-density transposon libraries utilising outward-oriented promoters identify mechanisms of action and resistance to antimicrobials
Source: FEMS Microbiol Lett. 2020 Nov 13;367(22):fnaa185. doi: 10.1093/femsle/fnaa185 (PMC7735965; doi:10.1093/femsle/fnaa185)
Supplement: fnaa185_Supplemental_Files [file fnaa185_supplemental_files.zip › SupplementaryMaterialMethods.docx]

**Supplementary Methods**

Transposon mutagenesis by *in vivo* transposition and library construction

Transposon mutagenesis was by *in vivo* transposition following electroporation with mini-transposon:transposase complexes. These were generated *in vitro* essentially as in (Goryshin *et al. Nat Biotechnol* 2000;**18**:97-100) by mixing 4 μL purified transposon (~500 ng); 8 μL purified Tn*5* transposase (~120 ng); 4 μL 100% glycerol and incubating at room temperature for 30 min. Complexes were stored at -20°C until use. Complexes were introduced into bacteria by electrotransformation to generate mutant libraries each containing a single transposon. Preparation of electrocompetent cells was briefly as follows - *E. coli*: 200 mL of exponential-phase L-broth culture (A600nm=0.2-0.3) was chilled on ice, bacteria harvested and washed three times in 100 mL ice-cold 10% glycerol, resuspended in a final volume of 500 µL ice-cold 10% glycerol and dispensed to 50 µL aliquots; *P. aeruginosa*: bacteria from 200 mL overnight L-broth culture were harvested, washed three times in room-temperature 300 mM sucrose, resuspended in a final volume of 1.5 mL in 300 mM sucrose and dispensed to 50 µL aliquots. Electroporation was at 2.4 kV, 200 Ω, 25 μF using 0.5 µL transposon:transposase complex followed by resuspension with 950 mL SOC buffer and incubation shaking at 37 °C for 1 h. The entire transformation was then plated onto L-agar with selection in 245 mm square assay plates and incubated overnight at 37 °C. Bacteria were harvested from the plates by washing with L-broth, glycerol was added to 15% (v/v) and aliquots were stored at -80°C. Typically at least 300000 colonies were harvested per transposon with transformations repeated to achieve these numbers. To test whether this method of library generation yielded mutants with a single transposon insertion, whole-genome sequencing (WGS) of 20 individual *E. coli* mutants was performed - 100% had a single transposon insertion. The number of individual mutants and insertion site frequency (**Table 1**) in the promoter libraries was estimated by determining the number of unique transposon sites in the pools by next generation sequencing (see below). Final combined library pools were made for each strain by mixing individual transposon pools normalised by A600nm measurement to contain approximately equal densities of each mutant.

Next generation sequencing for identification of TnAMICS1 insertion sites

Insertion sites were determined by next-generation sequencing on the IonTorrent (ThermoFisher) or Illumina platforms. Cell pellets were lysed by resuspension in 360 μL Lysis Buffer (Zymo Research) with 0.5 mm silica beads (~100 µl dry volume; Thistle Scientific) followed by shaking at 2000 r.p.m. for 20 min. DNA was then extracted from the lysate using a QIAcube (QIAGEN) and quantified spectrophotometrically. Genomic DNA was fragmented using the MuSeek Library Preparation Kit (ThermoFisher) - for Ion Torrent DNA was normalised to 5.56 ng/μL; for Illumina to 11.11 ng/μL - prior to fragmentation with the appropriate kit. For subsequent steps, PCR reactions were performed with NEBNext® High Fidelity PCR Master Mix. Fragmented DNA was then purified using KAPA Pure Beads (Roche) prior to addition of platform-specific adaptors by PCR: 98°C x 30 sec then 8 cycles of (98°C x 30 sec, 60°C x 60 sec, 72°C x 20 sec) with primer TnTra-01 (5’-TTGAGCGAATTCATTACCCTG-3’) followed by 10 cycles with the same conditions after addition of platform-specific primer - either MuSEEK/P1 (5’-CCACTACGCCTCCGCTTTCCTCTCTATGGGCAGTCGGTGATTTCGTGCGTCAGTTCA-3’) (IonTorrent) or MuSEEK/Illumina (5’-GTGACTGGAGTTCAGACGTGTGCTCTTCCGATCTGGCAGTCGGTGATTTCGTGCGTCAGTTCA-3’) - and a final extension of 72°C x 5 min. Reaction products were purified with KAPA Pure Beads (Roche) prior to addition of sample-specific barcodes (IonTorrent: IonXpress Barcode adaptors; Illumina: Combinatorial Dual Indexes) by PCR: 98°C x 30 sec then 12 cycles of (98°C x 10 sec, 62°C x 60 sec, 72°C x 20 sec), final extension 72°C x 5 min. For IonTorrent the reaction products were then size-selected (see later); for Illumina, products were purified with KAPA Pure Beads (Roche) prior to addition of indexing primers by PCR: 98°C x 30 sec then 12 cycles of (98°C x 10 sec, 62°C x 60 sec, 72°C x 20 sec), final extension 72°C x 5 min; fragments were then size-selected. Size selection was by KAPA Pure Beads (Roche), selecting for 150 b.p. (IonTorrent) or 250-350 b.p. Illumina.

Following size-selection, DNA was quantified using qPCR on a Roche Lightcycler and with – IonTorrent: Ion Library TaqMan^TM^ Quantitation Kit (ThemoFisher); Illumina: KAPA Library Quantification Kit (ABI Prism) (Roche). For IonTorrent, libraries were then normalised to 50 pM prior to loading on a P1 chip using the Ion Chef System and sequencing on an Ion Torrent Proton using P1 v3 sequencing chips; for Illumina, libraries were pooled using equimolar concentrations before diluting to a final concentration of 1.8pM using manufacturer’s protocol, 25% of PhiX was added and sequencing performed on an Illumina NextSeq 500 using the 75 cycle High Output kit with single-read, dual indexing sequencing.
