# Supplementary material for: High-density transposon libraries utilising outward-oriented promoters identify mechanisms of action and resistance to antimicrobials
Source: FEMS Microbiol Lett. 2020 Nov 13;367(22):fnaa185. doi: 10.1093/femsle/fnaa185 (PMC7735965; doi:10.1093/femsle/fnaa185)
Supplement: fnaa185_Supplemental_Files [file fnaa185_supplemental_files.zip › SupplementaryMaterialTables.docx]

**Supplementary material**

| [Fosfomycin] (xMIC_WT_) | No cpd | ¼ | ½ | ¾ | 1 | 2 | 4 |
| --- | --- | --- | --- | --- | --- | --- | --- |
| [Fosfomycin] (µg/mL) | 0 | 0.781 | 1.56 | 2.34 | 3.125 | 6.25 | 12.5 |
| Mapped insertion sites | ~370k | 10k/340k | ~50k | ~40k | 30k/290k | 80k/10k | 40k/10k |

**Table S1:** Number of mapped mini-transposon insertion sites for *E. coli* BW25113 libraries exposed to fosfomycin at the indicated concentrations with G6P at 25 μg/mL.

| [Fosfomycin] (xMIC_WT_) | No cpd | ¼ | ½ | ¾ | 1 | 2 | 4 |
| --- | --- | --- | --- | --- | --- | --- | --- |
| [Fosfomycin] (µg/mL) | 0 | 3.125 | 6.25 | 9.375 | 12.5 | 25 | 50 |
| Mapped insertion sites | ~450k | ~450k | ~375k | ~150k | ~50k | ~20k | ~20k |

**Table S2:** Number of mapped mini-transposon insertion sites for *P.* aeruginosa NCTC #11451 libraries exposed to fosfomycin at the indicated concentrations without G6P.

| Promoter | Source | Sequence (5’-3’) |
| --- | --- | --- |
| Outward-facing promoters to drive expression of chromosomal genes | | |
| PsPrrnB | Pa | TGTTCGCGGCAGCGGTTCGGTCCCGGAAGGGATTCGGAACGAAGCTTCGCCGAGGTGCTTGACAGCGAGTTTGAACGCTGTAGAATGCGCCTCCCGCTGATCGGAAGATGGTTTGAAGGTCAGCGCAAGCGGTTGAGTAGAAAAGAAAATTTTCGAAAATAACGCTTGACGGAACGAGAGGTTGCTGTAGAATGCGCGGCCTCGGTTGAGACGAAAGCCTTGACCAACTGCTCTTTAACAAGTCGAATCAAGCAATTCGTGTGGGTGCTTGTGATGTAAGACTGGTGATCGCAAGATTATCAGCAACACAAGTAACTCTGTGAATTCACAAGAGTTAATTGCGATTGCTGAGCCAAGTTTAGGGTTTTCTCAAAACCCAAGCAGTATTGAACTGAA |
| PrplJ | Ec | TGGCCTGAGCGCTTCTGTAAACTAATGCCTTTACGTGGGCGGTGATTTTGTCTACAATCTTACCCCCACGT |
| PrrnB | Ec | TTGCCTGAAAAATGAGCGAACGATAAAGTTTTTATCTTTTTCGCTTGTCAGGCCGGAATAACTCCCTATAATGCGCCACCACTGAC |
| Ptac* | Synthetic | TGTTGACAATTAATCATCGGCTCGTATAATGTGTGGAATTG |

**Table S3:** Promoters used in construction of mini-transposons. *contains -35 and -10 regions only, does not include the lacO operator for LacI repressor binding.
